# Telomerase Reverse Transcriptase Regulates microRNAs

**DOI:** 10.3390/ijms16011192

**Published:** 2015-01-06

**Authors:** Timo Lassmann, Yoshiko Maida, Yasuhiro Tomaru, Mami Yasukawa, Yoshinari Ando, Miki Kojima, Vivi Kasim, Christophe Simon, Carsten O. Daub, Piero Carninci, Yoshihide Hayashizaki, Kenkichi Masutomi

**Affiliations:** 1 RIKEN Omics Science Center, RIKEN Yokohama Institute, 1-7-22 Suehiro-cho, Tsurumi-ku, Yokohama 230-0045, Japan; E-Mails: lassmann@gsc.riken.jp (T.L.); tomaru@gsc.riken.jp (Y.T.); yando@gsc.riken.jp (Y.A.); miki1978@gsc.riken.jp (M.K.); simon@gsc.riken.jp (C.S.); daub@gsc.riken.jp (C.O.D.); carninci@riken.jp (P.C.); 2 Cancer Stem Cell Project, National Cancer Center Research Institute, 5-1-1 Tsukiji, Chuo-ku, Tokyo 104-0045, Japan; E-Mails: ymaida@ncc.go.jp (Y.M.); myasukaw@ncc.go.jp (M.Y.); vkasim@ncc.go.jp (V.K.); 3 Precursory Research for Embryonic Science and Technology (PREST), Japan Science and Technology Agency, 4-1-8 Honcho Kawaguchi, Saitama 332-0012, Japan

**Keywords:** telomerase reverse transcriptase, microRNA, RNA-dependent RNA polymerase, cancer

## Abstract

MicroRNAs are small non-coding RNAs that inhibit the translation of target mRNAs. In humans, most microRNAs are transcribed by RNA polymerase II as long primary transcripts and processed by sequential cleavage of the two RNase III enzymes, DROSHA and DICER, into precursor and mature microRNAs, respectively. Although the fundamental functions of microRNAs in RNA silencing have been gradually uncovered, less is known about the regulatory mechanisms of microRNA expression. Here, we report that telomerase reverse transcriptase (TERT) extensively affects the expression levels of mature microRNAs. Deep sequencing-based screens of short RNA populations revealed that the suppression of TERT resulted in the downregulation of microRNAs expressed in THP-1 cells and HeLa cells. Primary and precursor microRNA levels were also reduced under the suppression of TERT. Similar results were obtained with the suppression of either BRG1 (also called SMARCA4) or nucleostemin, which are proteins interacting with TERT and functioning beyond telomeres. These results suggest that TERT regulates microRNAs at the very early phases in their biogenesis, presumably through non-telomerase mechanism(s).

## 1. Introduction

MicroRNAs (miRNAs) are small non-coding RNAs that control diverse physiological and pathological processes through post-transcriptional regulation of protein synthesis. The basic steps in miRNA biogenesis are well understood [1]. After transcription, primary miRNA transcripts are cleaved by DROSHA, and the cleaved products are exported from the nucleus by XPO5 and, finally, cleaved by DICER. In addition to the canonical proteins, a number of post-transcriptional regulators of miRNA biogenesis have been discovered [2].

Somewhat less is known about the transcriptional regulation of miRNAs themselves. In humans, most of primary miRNAs (pri-miRNAs) are transcribed in an RNA polymerase II (Pol II)-dependent manner, and recent promoter analyses suggest transcriptional regulation of miRNAs might be similar to that of protein-coding genes. Indeed, some transcription factors have been shown to be important for pri-miRNA transcription, including MYC [3], STAT3 [4,5,6], TWIST1 [7,8] and NF-κB [9]; and epigenetic factors are also implicated in the regulation of pri-miRNA transcription [10]. However, the great majority of the regulatory mechanisms remain unknown.

Telomerase is a specialized ribonucleoprotein complex that elongates telomeres through its RNA-dependent DNA polymerase activity. Telomerase is composed of several proteins and a non-coding RNA, and the minimal essential components of the enzyme consist of telomerase reverse transcriptase (TERT) and its RNA template (*TERC*) [11]. Telomere maintenance via telomerase activity is indispensable for self-renewing cells, such as stem cells and cancers, in order to avoid replicative senescence. Recent evidence reveals that TERT also performs non-telomeric biological functions [12,13,14,15,16,17,18]. We recently identified that human TERT plays a role in RNA silencing [19,20]. We found that TERT interacts with BRG1 (also called SMARCA4) and nucleostemin (NS) [20,21] and exerts mammalian RNA-dependent RNA polymerase (RdRP) activity in mitotic cells. TERT-RdRP synthesizes double-stranded RNAs using single-stranded RNAs as templates, and the double-stranded RNAs are processed in a DICER-dependent manner into endogenous siRNAs that induce post-transcriptional gene silencing [19]; for example, TERT-RdRP mediates the production of endogenous siRNAs from a non-coding RNA (*RMRP*) using *RMRP* as a template [19]. TERT-RdRP also regulates transcriptional levels of heterochromatic regions in association with AGO2 [20]. These observations indicate that TERT may participate in the regulation of other classes of endogenous small RNA biogenesis, as well. To identify additional targets of TERT-based regulation and to understand the functional role of TERT in small RNA regulation, we conducted comprehensive screens of short RNA populations using next generation sequencing. We demonstrate that TERT largely participates in the regulation of miRNA biogenesis.

## 2. Results

### 2.1. Screening of Short RNAs Regulated by Telomerase Reverse Transcriptase (TERT)

Using human monocytic leukemia cell line THP-1, we conducted a broad screen targeting 5'-mono-phosphorylated, 5'-hydroxylated and 5'-capped short RNAs after the transfection of a gene-specific siRNA for TERT or a control siRNA. The experiment was carried out in duplicate, and the efficiency of the reduction of *TERT* by the specific siRNAs was at least 90% at the mRNA level (Figure S1a and Table S1).

We observed a high amount of transfer RNA (tRNA) fragments in all short RNA samples (Figures S2–S4), which is common when sequencing RNAs longer than 30 nucleotides (nt) [22]. Consequently, the number of available reads, or sequencing depth, was reduced for RNA classes other than tRNAs. As expected, the 5'-hydroxylated RNA fraction contained many ribosomal RNAs (rRNAs) [23] (Figure S4). Surprisingly, the ratio of miRNA population was apparently reduced by the suppression of TERT to the levels comparable to the suppression of either DICER or DROSHA (Figure S4 and Figure S5a). More specifically, 12 miRNAs were significantly downregulated (*p* < 0.05 after adjusting for multiple testing using the Benjamini and Hochberg method) upon TERT suppression (Figure S5b and Table S2). An additional 31 miRNAs were also reduced under TERT suppression, indicating that a total of 43 out of 104 miRNAs expressed in wild-type THP-1 cells were decreased by the suppression of TERT, while only six miRNAs showed a slight increase.

### 2.2. Validating TERT-Based miRNA Regulation

Since the number of reads available for miRNA sequencing was low when targeting a broad range of RNA lengths, we decided to repeat the sequencing experiment by specifically targeting the miRNA population (15–30 nt) for the validation of TERT-based regulation of miRNAs. Additionally, we expanded the experiments to both HeLa cells and THP-1 cells. As for HeLa cells, sequencing results of the cells infected with two independent shRNAs for TERT were individually compared with the results of the cells with a control shRNA (sh-GFP) (Figure S1b). For THP-1 cells, we compared our TERT-suppressed sample to a previously sequenced wild-type sample [24]. Due to the more restrictive RNA size selection, we obtained approximately eight-times more sequences per sample corresponding to known miRNAs compared to the initial screen (Figures S6, S7 and Table S1).

**Figure 1:**
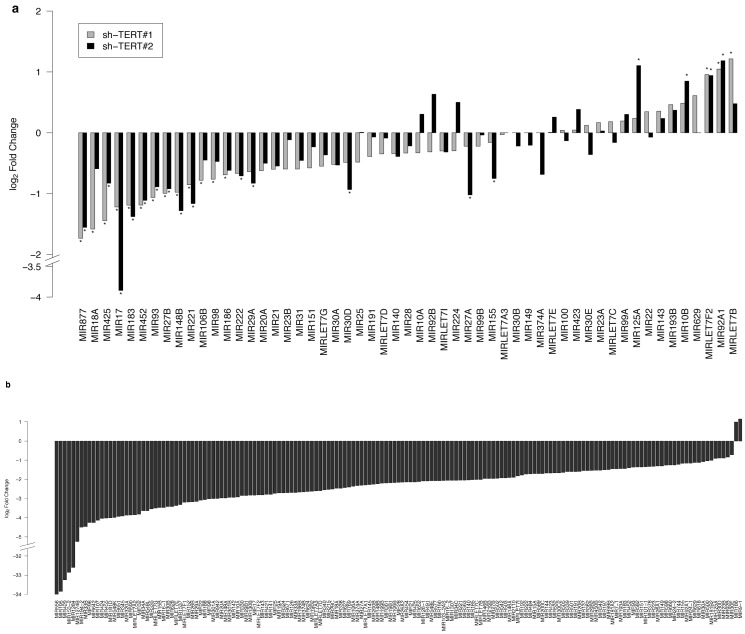
Mature miRNAs are regulated by TERT. Fold changes in miRNA expression measured by sequencing in HeLa cells (**a**) and THP-1 cells (**b**). Bars highlighted with asterisks represent statistically-significant changes. In HeLa cells, the changes were measured using sh-TERT#1 (gray) and sh-TERT#2 (black). In THP-1 cells, the changes were measured using a siRNA targeting TERT.

Concordantly with our broad screen, many miRNAs were downregulated upon TERT suppression (Figure 1). In HeLa cells, TERT suppression by two different shRNAs significantly downregulated a considerable number of miRNAs; 77 and 48 miRNAs, respectively (Figure 1a, Table S3 and Table S4). In comparison, only nine and eight miRNAs were upregulated by the shRNAs. Although there was only a little overlap between the miRNAs regulated by TERT in THP-1 broad screening and those in HeLa cells (Tables S2–S4), the results might reflect differences in the cell-type-specific steady-state profiles of miRNA expression. Similar to HeLa cells, a majority of the miRNAs was downregulated in THP-1 cells with TERT suppression (Figure 1b). Conclusively, TERT appears to act as a positive regulator of miRNA expression.

**Figure 2:**
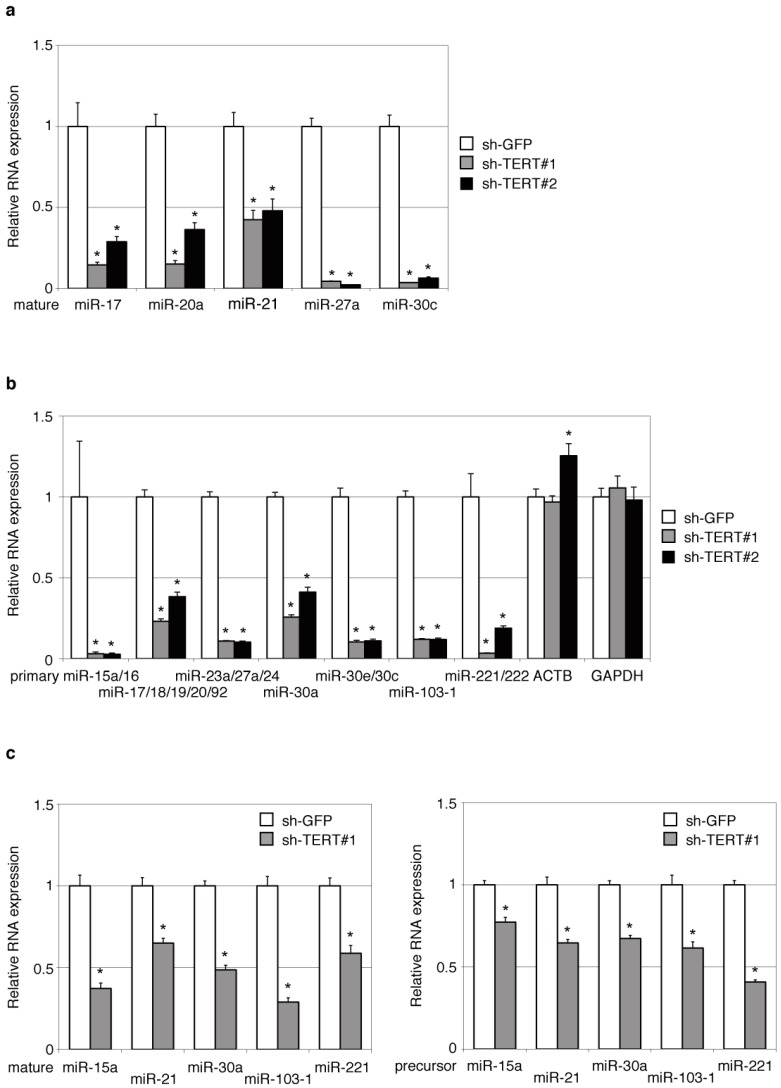
Mature miRNAs and their precursors are downregulated upon TERT suppression. The expression levels of primary, precursor and mature miRNAs in HeLa cells were analyzed with RT-qPCR under the suppression of TERT by sh-TERT#1 (gray) or sh-TERT#2 (black). The relative amount of the individual miRNAs was normalized to *U6*, and the mean expression levels of normalized sh-GFP (white) refer to one. (**a**,**b**) RT-qPCR of mature (**a**) and primary (**b**) miRNAs performed with total RNAs. The mRNA levels of *ACTB* and *GAPDH* were also examined with the same total RNAs; (**c**) RNAs smaller than 200 nt were used for RT-qPCR of mature (**left**) and precursor (**right**) miRNAs. Values represent the means ± SD for three independent experiments. Asterisks represent *p* < 0.05 compared with sh-GFP.

To further verify the deep sequencing findings, we quantified the expression levels of selected mature miRNAs under TERT suppression using quantitative RT-PCR (RT-qPCR). Corresponding with the deep sequencing findings (Figure 1), the RT-qPCR results indicated that mature miRNAs were apparently downregulated in both HeLa cells and THP-1 cells after reduction of TERT, irrespective of the suppression methods (Figure 2a and Figure S8). Taken together, these data confirm that TERT regulates mature miRNA levels in human cells.

### 2.3. Direct versus Indirect Regulation of miRNAs by TERT

Because TERT is a ribonucleoprotein that directly modifies physically-interacting RNAs [19], we investigated whether TERT regulates miRNA biogenesis directly or indirectly *in vivo*. We stably overexpressed a tandem affinity peptide (TAP)-tagged TERT protein in HeLa-S3 cells and isolated RNAs from immunoprecipitated TERT (Figure S9). Sequencing results of the TERT-interacting RNAs confirmed that human *TERC* and *RMRP* were among the most frequently isolated RNAs, as expected. However, there were only a few miRNAs in the precipitated RNA, and they were in mature forms with or without the parts of their adjacent stem-loop sequences (Table S5). Primary miRNA sequences were absent in the precipitated RNA. Since we expected to find the intermediates for many of the downregulated miRNAs at levels comparable to *RMRP*, we concluded that TERT does not physically interact with miRNA precursors.

To determine whether the reduction of mature miRNAs under TERT suppression occurred transcriptionally or post-transcriptionally, we conducted RT-qPCR targeting primary transcripts of the downregulated miRNAs. Surprisingly, the expression of primary miRNAs was clearly decreased in TERT-reduced HeLa cells for almost all miRNAs tested (Figure 2b and Figure S10). As expected, the precursor miRNAs of the tested miRNAs, the sequential products derived from primary miRNAs, were also reduced by the suppression of TERT (Figure 2c and Figure S10). Most of the primary miRNAs are transcribed by Pol II. If suppression of TERT modulates the essential functions of Pol II, it may affect the transcription of a wide variety of cellular RNAs, including both primary miRNAs and mRNAs. To ascertain the effect of TERT suppression on Pol II function, we performed RT-qPCR of representative Pol II products, mRNA of *ACTB* and *GAPDH*, with the same total RNAs used for primary miRNA analyses and found no remarkable decrease in the expression levels of these mRNAs (Figure 2b). The results indicated that TERT specifically regulates the transcripts of miRNA genes.

### 2.4. TERT May Regulate miRNAs in Cooperation with BRG1 and NS

**Figure 3:**
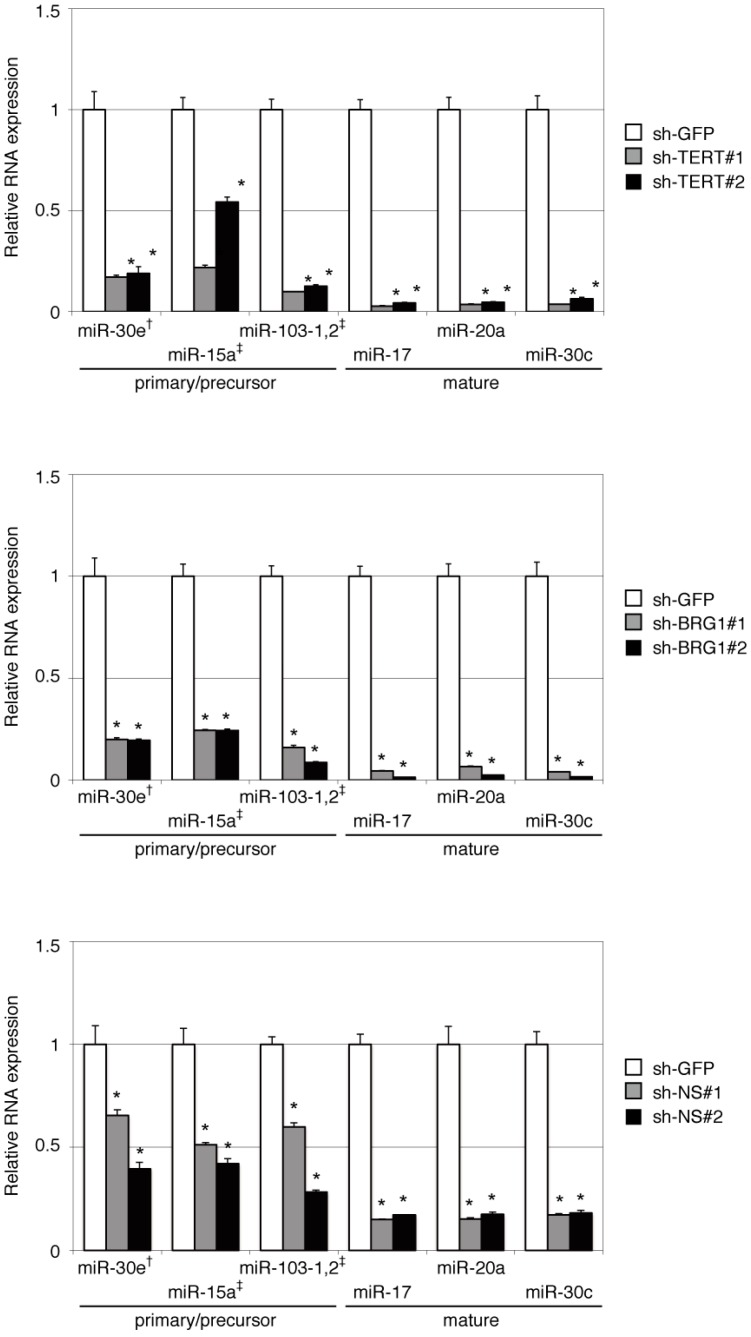
Comparable effects of TERT, BRG1 and NS in miRNA expression. The expression levels of mature miRNAs and miRNA precursors were examined with RT-qPCR upon suppression of TERT (**top**), BRG1 (**middle**) or NS (**bottom**) by gene-specific shRNAs. For miRNA precursors, there are the primers amplified primary form (^†^) or both primary and precursor forms (^‡^). The relative amount of the individual miRNAs was normalized to *U6*, and the mean expression levels in sh-GFP refer to one. Values represent the means ± SD for three independent experiments. Asterisks represent *p* < 0.05 compared with sh-GFP.

Recently, we and others have found that TERT interacts with BRG1 and NS and forms the complexes that regulate gene transcription and stem cell functions in both normal and malignant cells [21,25]. We have also found that the TERT/BRG1/NS (TBN) complex exerts an RdRP activity, and it is involved in heterochromatin maintenance [20]. To investigate whether the TBN complex contributes to the miRNA regulation, we conducted RT-qPCR of mature miRNAs and the precursors under the suppression of TERT, BRG1 or NS with gene-specific shRNAs [20,21]. The expression levels of mature miRNAs were reduced to comparable levels when knocking down TERT, BRG1 or NS (Figure 3). The primary and precursor miRNAs were also reduced by either BRG1 or NS suppression, just as seen under TERT suppression. These results support the hypothesis that TERT regulates miRNA transcription together with BRG1 and NS.

## 3. Discussion

Here, we demonstrate a novel function of TERT in the biogenesis of miRNAs. Deletion of TERT resulted in the dramatic decrease of most of the mature miRNAs, as well as their precursors, suggesting that TERT regulates miRNA levels at the early phase in miRNA processing. Primary miRNAs are transcribed by Pol II [26,27] or Pol III [28], and it was reported that the cleavage of pre-miRNAs from primary miRNAs occurs co-transcriptionally [29]. Because our results demonstrated that Pol II machinery stably transcribes cellular mRNAs under TERT suppression, it seems most likely that TERT regulates the transcriptional activity of miRNA gene promoters.

There are some reports analyzing putative promoter regions of miRNA genes; however, little is currently known about the transcriptional regulation of miRNA genes. Some researchers identified transcriptional start sites (TSSs) of miRNA genes based on the unique signature of histone modification restricted to TSS and/or the binding of Pol II [30,31,32,33]. The analysis of the identified miRNA promoters presented that the Pol II-transcribed miRNA promoters have similar characteristics as mRNA-encoding promoters. About 19% of the Pol II-transcribed miRNA promoters contain a TATA element; 21% have a TFIIB recognition element; and, 47% have an initiator; each ratio is concordant with that of mRNA promoters [32,34]. Potential binding motifs of some specific transcriptional factors are also mapped on miRNA promoters. Ozsolak *et al.* identified E-box elements, the DNA binding motifs of MYC, on 73 out of 175 human miRNA promoters [32], and Marson *et al.* [31] mapped the binding sites of the key ES cell transcriptional factors, Oct4, Sox2, Nanog and Tcf3, on about 20% of the miRNA promoters. The emerging evidence revealed that MYC upregulates many miRNAs [3,35,36,37,38,39], possibly via E-boxes. OCT4 and SOX2 are reported to regulate miRNA expression through binding to the putative promoter region of the miRNA [40,41]. We have previously reported that introduction of NS into TERT-immortalized fibroblast and cancer cells resulted in the significant increase of the expression of MYC, OCT4 and SOX2 [21]. According to our findings, it seems likely that TERT regulates miRNA transcription through induction of transcriptional factors, at least in part.

Wnt signaling and NF-κB signaling play important roles in both development and tumorigenesis. In the canonical Wnt pathway, the β-catenin-TCF complex promotes transcriptional activation of Wnt target genes. BRG1 is a SWI/SNF-related ATP-dependent chromatin-remodeling factor, which interacts with β-catenin-TCF complex in the transcriptional regulation. Intriguingly, we have reported that NS interacts with both TERT and BRG1 and increases the levels of activated β-catenin [21]. Park *et al.* also reported that TERT interacts with BRG1, and TERT-BRG1 is involved in the canonical Wnt/β-catenin pathway [25]. It is emerging that Wnt/β-catenin pathway regulates the transcription of miRNA genes. Fujita *et al.* [42] reported that phorbol 12-myristate 13-acetate (PMA) induced miR-21 upregulation, in which BRG1 facilitates transcriptional activation in corporation with AP-1. Mallappa *et al.* [43] also reported that the deficiencies in BRG1 equate to the deficiency of DICER, and BRG1 was required for the MyoD-dependent transcription of myogenic miRNAs. *MYC* protooncogene is known to be the target of the β-catenin-TCF complex [44,45]. In our experiments, suppression of either BRG1 or NS reduced miRNA levels comparable to that of TERT suppression, and we feel it is likely that the TBN complex is responsible for the TERT-based miRNA regulation, partly via the Wnt/β-catenin pathway. At this stage, however, it is unclear whether the TBN complex directly binds to miRNA promoters or whether the regulation is indirect; in addition, it is also uncertain which miRNA promoters would be regulated directly (or indirectly) by the TBN complex. Further studies, including ectopic expression of the TBN components or chromatin immunoprecipitation by the TBN complex, will be required to fully understand the regulatory mechanisms. NF-κB is a transcriptional factor, and NF-κB-dependent induction of miRNAs has been reported [9]. Since TERT binds to the NF-κB p65 subunit and upregulates NF-κB-dependent transcriptions [46], TERT may regulate a part of miRNA expression through NF-κB.

In addition to transcription factors, CpG islands are found in about 60% of the Pol II-transcribed miRNA promoters, and the histone modification patterns of the promoters are indistinguishable from those of mRNA-encoding promoters [32,33]; therefore, it is now believed that the transcriptional activity of miRNA promoters is regulated epigenetically, as well. The TBN complex is involved in the heterochromatic maintenance of repetitive sequences, such as *Satellite I* at the centromere and transposable element *LINE1* [20], and it may be possible that the complex contributes to the epigenetic regulation of other genetic elements, including miRNA genes.

Consistent with our study, Drevytska *et al.* [47] recently reported a positive correlation between TERT expression levels and several miRNA levels. They induced TERT expression in the cultured neonatal rat cardiomyocytes via anoxia-reoxygenation modeling and found the increased expression of immature (pri- and pre-) miR-1 and miR-29a, as well as mature miR-1. Conversely, siRNA-mediated TERT suppression decreased mature miR-21, miR-29a and miR-208a. Although they only focused on and tested miRNAs predominantly specific for the heart (miR-1, miR-21, miR-29a and miR-208a), their findings suggest that miRNA regulation by TERT may be a conserved gene regulatory mechanism across species.

## 4. Experimental Section

### 4.1. Cell Culture

THP-1 cells were cultured in RPMI1640 (Invitrogen, Carlsbad, CA, USA) supplemented with 10% FBS, 10 mM HEPES (Invitrogen), 1 mM sodium pyruvate (Invitrogen) and 50 μM 2-mercaptoethanol (Invitrogen) [24]. HeLa cells and HeLa-S3 cells were cultured in DMEM (Wako, Osaka, Japan) supplemented with 10% FBS.

### 4.2. siRNA Transfection, RNA Extraction and Expression Analysis by RT-qPCR

siRNA transfection and expression analysis of the target genes were performed as described previously [24]. The suppression of TERT in THP-1 cells was performed with the specific siRNA (sense: 5'-GAGCAAGUUGCAAAGCAUUTT-3', antisense: 5'-AAUGCUUUGCAACUUGCUCTT-3'). Reverse transfection of 1 × 10^6^ cells in a 60-mm cell culture dish was performed with 20 nM (final concentration) of each siRNA, Opti-MEM (Invitrogen) and 1.6 μg/mL (final concentration) of Lipofectamine 2000 (Invitrogen), according to the manufacturer’s instructions. RNAs were extracted 72 h after transfection with TRIzol (Invitrogen) and the FastPure RNA kit (TaKaRa, Otsu, Japan), according to the modified manufacturer’s instructions. Expression levels of siRNA target genes in the cells treated with the specific siRNAs or the calibrator negative control siRNA were estimated by RT-qPCR in triplicate with the specific primer sets: 5'-AGCTGACGTGGAAGATGAGC-3' and 5'-ATCAGCCAGTGCAGGAACTT-3' for TERT, 5'-TCTGTACATTCAAAAGAAAGAGATTC-3' and 5'-CAGGGTCCCAGAACTACCAA-3' for DICER and 5'-CACAGGAATTAGGCACAGCA-3' and 5'-GGGAGACTGTGATCCGGTAG-3' for DROSHA. The procedures for RT-qPCR were essentially as described [48]. Reverse transcriptase reactions were performed with SuperScript VILO (Invitrogen), and qPCRs were performed with TAKARA SYBR Premix Ex Taq (TaKaRa), according to the manufacturer’s instructions. Individual mature miRNA expression analysis was performed with the miScript system (Qiagen, Hilden, Germany) according to the manufacturer’s instructions. Expression levels of mature miRNAs in the cells treated with TERT-specific siRNAs or the calibrator negative control siRNA were estimated by RT-qPCR in triplicate with the specific miScript primers (Qiagen).

### 4.3. shRNA Transduction, RNA Extraction and Expression Analysis by RT-qPCR

shRNAs targeting TERT and BRG1 were constructed by The RNAi Consortium, and shRNAs targeting NS were constructed in-house using the pLKO.1-puro vector. The target sequence of each shRNA was as follows [21]; sh-TERT#1 (clone ID: TRCN0000219794), 5'-CCTGCGTTTGGTGGATGATTT-3': sh-TERT#2 (clone ID: TRCN0000179552), 5'-GACATGGAGAACAAGCTGTTT-3': sh-BRG1#1 (clone ID: TRCN0000015549), 5'-CCCGTGGACTTCAAGAAGATA-3': sh-BRG1#2 (clone ID: TRCN0000015552), 5'-CGGCAGACACTGTGATCATTT-3': sh-NS#1, 5'-GCACTGTCTGAGGAGACTACA-3': sh-NS#2, 5'-GGAGGCTCTTCTTAGGGAAGC-3'. sh-GFP was used as the control. To suppress TERT expression in HeLa cells, amphotropic lentiviruses were created with the pLKO.1-puro vectors, and the viral sup was used for infection. After infection, the cells were selected using puromycin (2 µg/mL) for three days. Total RNAs were extracted with TRIzol (Invitrogen) and a FastPure RNA kit (TaKaRa) according to the manufacture’s protocols. Small RNA fractions were extracted using mirVana miRNA Isolation Kit (Ambion, Austin, TX, USA), followed by ethanol precipitation at −20 °C overnight. Reverse transcriptions were performed by using miScript Reverse Transcription Kit (Qiagen), and RT-qPCR was performed by using LightCycler 480 SYBR Green I Master (Roche Diagnostics, Mannheim, Germany), according to the manufacturer’s protocol. miScript primers (Qiagen) were used for the detection of mature miRNAs. Primer pairs used for primary transcripts of miRNAs and precursor miRNAs were previously designed [49,50]. *U6* was used as the reference. All reactions were performed in triplicate.

### 4.4. Library Construction and Sequencing

RNAs of 15 to 50 nt in length were included in the initial screen with THP-1 cells to explore changes in a broad range of small RNA classes. For efficiency, RNAs from individual samples were labeled using barcode sequences, and six samples were pooled into one library to be sequenced together in one lane of the Illumina Genome Analyzer (Illumina, San Diego, CA, USA) for the large-scale screening of RNA populations. The procedures for the short RNA library constructions were essentially as described in [51]. The library for the deep sequencing of miRNAs was constructed using the Small RNA Sample Kit (Illumina), according to the manufacturer’s instructions. Libraries were sequenced using the Genome Analyzer (Illumina).

RNA fractionation was performed as described previously [24]. Cell lysis homogenization and extraction of total RNA were performed with TRIzol (Invitrogen), according to the manufacturer’s instructions. Then FastPure RNA kit (TaKaRa) was used for RNA purification and fractionation, according to modified manufacturer’s instructions. After phase separation of TRIzol, the aqueous phase was mixed with the same amount of 70% ethanol and flowed through a filter cartridge to capture RNA longer than 200 nt. The flow through was then mixed with ethanol to obtain a 50% final concentration and flowed through a new filter cartridge to capture RNAs smaller than 200 nt. After RNA binding to the filter cartridge, each bound RNA was washed three times with 700 μL of treble ethanol diluted wash buffer and eluted with elution buffer.

Three micrograms of the short RNA fraction were used for each library construction after denaturation at 65 °C for 5 min. For a capped library, the phosphatase reaction and decapping reaction were done before sequence linker ligation. The phosphatase reaction was performed at 37 °C for 2 h in 30 μL reaction volume with 30 units of Antarctic phosphatase (NEB, Ipswich, MA, USA), according to the manufacturer’s instructions. Tobacco acid pyrophosphatase reaction for removing the CAP structure was performed at 37 °C for 1 h with 6 units of tobacco acid pyrophosphatase (EPICENTRE, Madison, WI, USA), according to the manufacturer’s instructions. For a hydroxylated library, the phosphorylation reaction was done before linker ligation. The T4 polynucleotide kinase reaction for phosphorylation was performed at 37 °C for 30 min in 100 μL reaction volume with 40 units of T4 polynucleotide kinase (NEB), according to the manufacturer’s instructions. After each enzyme reaction, products were purified by phenol:chloroform:isoamyl alcohol (25:24:1) extraction and ethanol precipitation.

After pretreatment, each short RNA was ligated with a PAGE purified RNA-DNA hybrid 5' adaptor tag containing a sample-specific four-nucleotide tag sequence (5'-ACAGGTTCAGAGTTCTACAGXXXXA-OH-3') and 3' adaptor mix containing 3' biotinylation and 5' phosphorylation (5'-phosphate-uuuTCGTATGCCGTCTTCTGCTTG-Biotin-3') with T4 RNA ligase (TaKaRa) for 16 h at 15 °C. Tag-ligated short RNAs were purified with PAGE extraction, and an RT-PCR reaction was performed for cDNA synthesis with reverse transcriptase and primer L1 (5'-CAAGCAGAAGACGGCATACGA-3'). The subsequent PCR reaction was performed with the same primer L1 and primer U3 (5'-ACAGGTTCAGAGTTCTACAG-3'). RT products were calibrated to determine the ratio of products derived from individual knockdown and pretreated samples in the library and purified by PAGE extraction.

cDNA derived from RT-PCR was amplified by PCR to add sequencing of the oligonucleotide sequence with primer L1 and primer U1 (5'-AATGATACGGCGACCACGACAGGTTCAGAGTTCTACAG-3') and purified with PAGE extraction.

Purified PCR products for sequencing were sequenced using Genome Analyzer (Illumina) with the sequencing primer (5'-CGGCGACCACGACAGGTTCAGAGTTCTACAG-3'), according to the manufacturer’s instructions.

### 4.5. Bioinformatics

In total, 61.4 million sequences were obtained in the initial screening experiment and an additional 23.6 million in the experiment focused on the miRNA fraction. We applied the program TagDust [52] to the sequenced libraries to remove all captured siRNA and adaptor sequences used in the experiment. Furthermore, we removed all sequences that were found less than 10 times in all experiments. All obtained reads were mapped to the human genome (hg19) using Delve. In brief, Delve uses a pair hidden Markov model (pHMM) to iteratively map reads to the genome and to estimate position-dependent error probabilities. After all error probabilities are estimated, individual reads are placed into a single position on the genome, where the alignment has the highest probability of being true according to the pHMM model. Phred-scaled mapping qualities, reflecting the likelihood of the alignment at a genome position, are also reported. Reads mapping with a quality of less than 10 (<90% chance of true) were discarded.

To assign reads to individual miRNA loci, we intersected mapping locations with the genomic boundaries of known miRNAs obtained from GENCODE (http://www.gencodegenes.org/) using BEDtools [53]. We used the same approach with additional annotation categories to obtain a global pre-library overview of what was sequenced (Figures S2–S4, S6 and S7).

To detect differentially expressed reads in our libraries, we first summed the number of reads mapping to each miRNA and applied the R package edgeR [54]. We used the exact test to detect differences in read counts exactly as described in the documentation using the short RNA example. In the absence of replicas, we estimated the read dispersion by treating both the control and test libraries as replicas. Differentially-expressed miRNAs with a false discovery rate less than 5% alongside their concentration and fold-changes are listed in Tables S2–S4 for all comparisons.

In Figure S5a, the sequences were analyzed with TagDust and mapped to the human genome (hg18) using the program, Nexalign [55].

### 4.6. Purification of TERT Complexes and Isolation of RNAs

Purification of TERT complexes was performed as described previously [19]. For this, 2 × 10^8^ HeLa-S3 cells expressing or lacking (control) TAP-TERT were lysed in 5 mL of Lysis Buffer A (LBA; 20 mM Tris-HCl pH 7.4, 150 mM NaCl, 0.5% NP-40, 0.1 mM DTT) and incubated for 30 min on ice. The lysate was then centrifuged at 16,000× *g* for 20 min at 4 °C. The supernatant was incubated with the anti-FLAG (M2) antibody conjugated agarose (SIGMA, Saint Louis, MO, USA) overnight at 4 °C. The beads were washed 3 times with LBA and eluted with 3× FLAG peptide (150 ng/μL). The resulting elution was incubated with protein A agarose beads (PIERCE, Rockford, IL, USA) and an anti-HA antibody (F7; Santa Cruz, Santa Cruz, CA, USA) for 4 h at 4 °C. The beads were washed 3 times with LBA, and RNA was isolated using TRIzol (Invitrogen). RNA samples prepared in this manner were analyzed with an Experion capillary electrophoresis device (Bio-Rad, Hercules, CA, USA) to visualize RNA species. Twenty percent of the RNA was used for RT-qPCR, and the remaining 80% was analyzed by deep sequencing. RT-qPCR was performed with a LightCycler 480 II (Roche Diagnostics), according to the manufacturer’s protocols. The TaqMan Gene Expression Assay system was used for human *TERC* analysis. The expression levels of *RMRP* were detected using the following primers and probe [19]; forward primer (5'-GAGAGTGCCACGTGCATACG-3'), reverse primer (5'-CTCAGCGGGATACGCTTCTT-3'), VIC-labeled TaqMan MGB probe (5'-ACGTAGACATTCCCC-3').

## 5. Conclusions

Our comprehensive analysis clearly demonstrated TERT-based positive regulation of miRNAs in human cells. Involvement of BRG1 and NS, as well as TERT in the regulation indicates that the TBN complex, and not the telomerase complex, may be in charge of this novel function of TERT. It is now speculated that TERT plays multiple roles in both physiological and pathological processes, not only by affecting telomere homeostasis, but also by regulating small RNA homeostasis, including both endogenous siRNA and miRNA. The detailed mechanisms of TERT-mediated miRNA regulation will be uncovered in future studies.

## Supplementary Materials

Supplementary materials can be found at http://www.mdpi.com/1422-0067/16/01/1192/s1.

## References

[1] Bartel D.P. (2004). MicroRNAs: Genomics, biogenesis, mechanism, and function. Cell.

[2] Siomi H., Siomi M.C. (2010). Posttranscriptional regulation of microRNA biogenesis in animals. Mol. Cell.

[3] O’Donnell K.A., Wentzel E.A., Zeller K.I., Dang C.V., Mendell J.T. (2005). c-Myc-regulated microRNAs modulate E2F1 expression. Nature.

[4] Loffler D., Brocke-Heidrich K., Pfeifer G., Stocsits C., Hackermuller J., Kretzschmar A.K., Burger R., Gramatzki M., Blumert C., Bauer K. (2007). Interleukin-6 dependent survival of multiple myeloma cells involves the Stat3-mediated induction of microRNA-21 through a highly conserved enhancer. Blood.

[5] Brock M., Trenkmann M., Gay R.E., Michel B.A., Gay S., Fischler M., Ulrich S., Speich R., Huber L.C. (2009). Interleukin-6 modulates the expression of the bone morphogenic protein receptor type II through a novel STAT3-microRNA cluster 17/92 pathway. Circ. Res..

[6] Iliopoulos D., Jaeger S.A., Hirsch H.A., Bulyk M.L., Struhl K. (2010). STAT3 activation of miR-21 and miR-181b-1 via PTEN and CYLD are part of the epigenetic switch linking inflammation to cancer. Mol. Cell.

[7] Ma L., Teruya-Feldstein J., Weinberg R.A. (2007). Tumour invasion and metastasis initiated by microRNA-10b in breast cancer. Nature.

[8] Lee Y.B., Bantounas I., Lee D.Y., Phylactou L., Caldwell M.A., Uney J.B. (2009). Twist-1 regulates the miR-199a/214 cluster during development. Nucleic Acids Res..

[9] Taganov K.D., Boldin M.P., Chang K.J., Baltimore D. (2006). NF-κB-dependent induction of microRNA miR-146, an inhibitor targeted to signaling proteins of innate immune responses. Proc. Natl. Acad. Sci. USA.

[10] Baer C., Claus R., Plass C. (2013). Genome-wide epigenetic regulation of miRNAs in cancer. Cancer Res..

[11] Weinrich S.L., Pruzan R., Ma L., Ouellette M., Tesmer V.M., Holt S.E., Bodnar A.G., Lichtsteiner S., Kim N.W., Trager J.B. (1997). Reconstitution of human telomerase with the template RNA component hTR and the catalytic protein subunit hTRT. Nat. Genet..

[12] Gonzalez-Suarez E., Samper E., Ramirez A., Flores J.M., Martin-Caballero J., Jorcano J.L., Blasco M.A. (2001). Increased epidermal tumors and increased skin wound healing in transgenic mice overexpressing the catalytic subunit of telomerase, mTERT, in basal keratinocytes. EMBO J..

[13] Artandi S.E., Alson S., Tietze M.K., Sharpless N.E., Ye S., Greenberg R.A., Castrillon D.H., Horner J.W., Weiler S.R., Carrasco R.D. (2002). Constitutive telomerase expression promotes mammary carcinomas in aging mice. Proc. Natl. Acad. Sci. USA.

[14] Masutomi K., Possemato R., Wong J.M., Currier J.L., Tothova Z., Manola J.B., Ganesan S., Lansdorp P.M., Collins K., Hahn W.C. (2005). The telomerase reverse transcriptase regulates chromatin state and DNA damage responses. Proc. Natl. Acad. Sci. USA.

[15] Sarin K.Y., Cheung P., Gilison D., Lee E., Tennen R.I., Wang E., Artandi M.K., Oro A.E., Artandi S.E. (2005). Conditional telomerase induction causes proliferation of hair follicle stem cells. Nature.

[16] Lee J., Sung Y.H., Cheong C., Choi Y.S., Jeon H.K., Sun W., Hahn W.C., Ishikawa F., Lee H.W. (2008). TERT promotes cellular and organismal survival independently of telomerase activity. Oncogene.

[17] Low K.C., Tergaonkar V. (2013). Telomerase: Central regulator of all of the hallmarks of cancer. Trends. Biochem. Sci..

[18] Li Y., Tergaonkar V. (2014). Noncanonical functions of telomerase: Implications in telomerase-targeted cancer therapies. Cancer Res..

[19] Maida Y., Yasukawa M., Furuuchi M., Lassmann T., Possemato R., Okamoto N., Kasim V., Hayashizaki Y., Hahn W.C., Masutomi K. (2009). An RNA-dependent RNA polymerase formed by TERT and the RMRP RNA. Nature.

[20] Maida Y., Yasukawa M., Okamoto N., Ohka S., Kinoshita K., Totoki Y., Ito T.K., Minamino T., Nakamura H., Yamaguchi S. (2014). Involvement of telomerase reverse transcriptase in heterochromatin maintenance. Mol. Cell Biol..

[21] Okamoto N., Yasukawa M., Nguyen C., Kasim V., Maida Y., Possemato R., Shibata T., Ligon K.L., Fukami K., Hahn W.C. (2011). Maintenance of tumor initiating cells of defined genetic composition by nucleostemin. Proc. Natl. Acad. Sci. USA.

[22] Kawaji H., Nakamura M., Takahashi Y., Sandelin A., Katayama S., Fukuda S., Daub C.O., Kai C., Kawai J., Yasuda J. (2008). Hidden layers of human small RNAs. BMC Genomics.

[23] Hannon G.J., Maroney P.A., Branch A., Benenfield B.J., Robertson H.D., Nilsen T.W. (1989). Accurate processing of human pre-rRNA *in vitro*. Mol. Cell Biol..

[24] Burroughs A.M., Ando Y., de Hoon M.J., Tomaru Y., Nishibu T., Ukekawa R., Funakoshi T., Kurokawa T., Suzuki H., Hayashizaki Y. (2010). A comprehensive survey of 3' animal miRNA modification events and a possible role for 3' adenylation in modulating miRNA targeting effectiveness. Genome Res..

[25] Park J.I., Venteicher A.S., Hong J.Y., Choi J., Jun S., Shkreli M., Chang W., Meng Z., Cheung P., Ji H. (2009). Telomerase modulates Wnt signalling by association with target gene chromatin. Nature.

[26] Cai X., Hagedorn C.H., Cullen B.R. (2004). Human microRNAs are processed from capped, polyadenylated transcripts that can also function as mRNAs. RNA.

[27] Lee Y., Kim M., Han J., Yeom K.H., Lee S., Baek S.H., Kim V.N. (2004). MicroRNA genes are transcribed by RNA polymerase II. EMBO J..

[28] Borchert G.M., Lanier W., Davidson B.L. (2006). RNA polymerase III transcribes human microRNAs. Nat. Struct. Mol. Biol..

[29] Morlando M., Ballarino M., Gromak N., Pagano F., Bozzoni I., Proudfoot N.J. (2008). Primary microRNA transcripts are processed co-transcriptionally. Nat. Struct. Mol. Biol..

[30] Fujita S., Iba H. (2008). Putative promoter regions of miRNA genes involved in evolutionarily conserved regulatory systems among vertebrates. Bioinformatics.

[31] Marson A., Levine S.S., Cole M.F., Frampton G.M., Brambrink T., Johnstone S., Guenther M.G., Johnston W.K., Wernig M., Newman J. (2008). Connecting microRNA genes to the core transcriptional regulatory circuitry of embryonic stem cells. Cell.

[32] Ozsolak F., Poling L.L., Wang Z., Liu H., Liu X.S., Roeder R.G., Zhang X., Song J.S., Fisher D.E. (2008). Chromatin structure analyses identify miRNA promoters. Genes Dev..

[33] Corcoran D.L., Pandit K.V., Gordon B., Bhattacharjee A., Kaminski N., Benos P.V. (2009). Features of mammalian microRNA promoters emerge from polymerase II chromatin immunoprecipitation data. PLoS One.

[34] Carninci P., Sandelin A., Lenhard B., Katayama S., Shimokawa K., Ponjavic J., Semple C.A., Taylor M.S., Engstrom P.G., Frith M.C. (2006). Genome-wide analysis of mammalian promoter architecture and evolution. Nat. Genet..

[35] Dews M., Homayouni A., Yu D., Murphy D., Sevignani C., Wentzel E., Furth E.E., Lee W.M., Enders G.H., Mendell J.T. (2006). Augmentation of tumor angiogenesis by a Myc-activated microRNA cluster. Nat. Genet..

[36] Lin C.H., Jackson A.L., Guo J., Linsley P.S., Eisenman R.N. (2009). Myc-regulated microRNAs attenuate embryonic stem cell differentiation. EMBO J..

[37] Kim J.W., Mori S., Nevins J.R. (2010). Myc-induced microRNAs integrate Myc-mediated cell proliferation and cell fate. Cancer Res..

[38] Mestdagh P., Fredlund E., Pattyn F., Schulte J.H., Muth D., Vermeulen J., Kumps C., Schlierf S., de Preter K., van Roy N. (2010). MYCN/c-MYC-induced microRNAs repress coding gene networks associated with poor outcome in MYCN/c-MYC-activated tumors. Oncogene.

[39] Smith K.N., Singh A.M., Dalton S. (2010). Myc represses primitive endoderm differentiation in pluripotent stem cells. Cell Stem Cell.

[40] Card D.A., Hebbar P.B., Li L., Trotter K.W., Komatsu Y., Mishina Y., Archer T.K. (2008). Oct4/Sox2-regulated miR-302 targets cyclin D1 in human embryonic stem cells. Mol. Cell Biol..

[41] Liu H., Deng S., Zhao Z., Zhang H., Xiao J., Song W., Gao F., Guan Y. (2011). Oct4 regulates the miR-302 cluster in P19 mouse embryonic carcinoma cells. Mol. Biol. Rep..

[42] Fujita S., Ito T., Mizutani T., Minoguchi S., Yamamichi N., Sakurai K., Iba H. (2008). miR-21 Gene expression triggered by AP-1 is sustained through a double-negative feedback mechanism. J. Mol. Biol..

[43] Mallappa C., Nasipak B.T., Etheridge L., Androphy E.J., Jones S.N., Sagerstrom C.G., Ohkawa Y., Imbalzano A.N. (2010). Myogenic microRNA expression requires ATP-dependent chromatin remodeling enzyme function. Mol. Cell Biol..

[44] He T.C., Sparks A.B., Rago C., Hermeking H., Zawel L., da Costa L.T., Morin P.J., Vogelstein B., Kinzler K.W. (1998). Identification of c-MYC as a target of the APC pathway. Science.

[45] Van de Wetering M., Sancho E., Verweij C., de Lau W., Oving I., Hurlstone A., van der Horn K., Batlle E., Coudreuse D., Haramis A.P. (2002). The β-catenin/TCF-4 complex imposes a crypt progenitor phenotype on colorectal cancer cells. Cell.

[46] Ghosh A., Saginc G., Leow S.C., Khattar E., Shin E.M., Yan T.D., Wong M., Zhang Z., Li G., Sung W.K. (2012). Telomerase directly regulates NF-κB-dependent transcription. Nat. Cell Biol..

[47] Drevytska T.I., Nagibin V.S., Gurianova V.L., Kedlyan V.R., Moibenko A.A., Dosenko V.E. (2014). Silencing of TERT decreases levels of miR-1, miR-21, miR-29a and miR-208a in cardiomyocytes. Cell Biochem. Funct..

[48] Tomaru Y., Nakanishi M., Miura H., Kimura Y., Ohkawa H., Ohta Y., Hayashizaki Y., Suzuki M. (2009). Identification of an inter-transcription factor regulatory network in human hepatoma cells by Matrix RNAi. Nucleic Acids Res..

[49] Jiang J., Lee E.J., Gusev Y., Schmittgen T.D. (2005). Real-time expression profiling of microRNA precursors in human cancer cell lines. Nucleic Acids Res..

[50] Suzuki H.I., Yamagata K., Sugimoto K., Iwamoto T., Kato S., Miyazono K. (2009). Modulation of microRNA processing by p53. Nature.

[51] Taft R.J., Glazov E.A., Cloonan N., Simons C., Stephen S., Faulkner G.J., Lassmann T., Forrest A.R., Grimmond S.M., Schroder K. (2009). Tiny RNAs associated with transcription start sites in animals. Nat. Genet..

[52] Lassmann T., Hayashizaki Y., Daub C.O. (2009). TagDust—A program to eliminate artifacts from next generation sequencing data. Bioinformatics.

[53] Quinlan A.R., Hall I.M. (2010). BEDTools: A flexible suite of utilities for comparing genomic features. Bioinformatics.

[54] Robinson M.D., McCarthy D.J., Smyth G.K. (2010). edgeR: A Bioconductor package for differential expression analysis of digital gene expression data. Bioinformatics.

[55] Lassmann T. Nexalign. http://134.160.84.27/osc/english/dataresource/index.html.

